# Cordycepin disrupts leukemia association with mesenchymal stromal cells and eliminates leukemia stem cell activity

**DOI:** 10.1038/srep43930

**Published:** 2017-03-07

**Authors:** Shu-Man Liang, Yi-Jhu Lu, Bor-Sheng Ko, Yee-Jee Jan, Song-Kun Shyue, Shaw-Fang Yet, Jun-Yang Liou

**Affiliations:** 1 Institute of Cellular and System Medicine, National Health Research Institutes, Zhunan 350, Taiwan; 2 Department of Internal Medicine, National Taiwan University Hospital, Taipei 100, Taiwan; 3 Department of Pathology and Laboratory Medicine, Taichung Veterans General Hospital, Taichung 407, Taiwan; 4 Institute of Biomedical Sciences, Academia Sinica, Taipei 115, Taiwan; 5 Graduate Institute of Basic Medical Science, China Medical University, Taichung 404, Taiwan

## Abstract

Maintaining stemness of leukemic stem cells (LSCs) and reciprocal interactions between leukemia and stromal cells support leukemic progression and resistance to chemotherapy. Targeting the niche-based microenvironment is thus a new approach for leukemia therapy. Cordycepin is an analogue of adenosine and has been suggested to possess anti-leukemia properties. However, whether cordycepin influences association of leukemia and mesenchymal stromal cells has never been investigated. Here we show that cordycepin reduces CD34^+^CD38^−^ cells in U937 and K562 cells and induces Dkk1 expression via autocrine and paracrine regulation in leukemia and mesenchymal stromal/stem cells (MSCs). Cordycepin suppresses cell attachment of leukemia with MSCs and downregulates N-cadherin in leukemia and VCAM-1 in MSCs. Moreover, incubation with leukemic conditioned media (CM) significantly induces IL-8 and IL-6 expression in MSCs, which is abrogated by cordycepin. Suppression of leukemic CM-induced VCAM-1 and IL-8 by cordycepin in MSCs is mediated by impairing NFκB signaling. Finally, cordycepin combined with an adenosine deaminase inhibitor prolongs survival in a leukemic mouse model. Our results indicate that cordycepin is a potential anti-leukemia therapeutic adjuvant via eliminating LSCs and disrupting leukemia-stromal association.

The wnt/β-catenin signal pathway contributes to the development of leukemia stem cells (LSCs) and disease progression in both acute myeloid leukemia (AML)^1^,^2^,^3^ and chronic myeloid leukemia (CML)^4^,^5^,^6^,^7^,^8^,^9^. An increase of β-catenin is a poor prognosis predictor in AML and is essential for the survival of LSCs that are insensitive to kinase inhibitors in CML^2^,^9^. Moreover, an association with the hematopoietic microenvironment and wnt signaling are required during the progression of AML^10^. Attenuation of wnt/β-catenin signaling in LSCs is thus a potential therapeutic strategy for leukemia treatment^3^,^4^. Wnt ligands bind to the transmembrane Frizzed receptor and receptor protein LRP5/6 (low-density lipoprotein receptor related protein 5 and 6) to activate β-catenin and its downstream gene expression^11^,^12^. Dickkopf-1 (Dkk-1) is a secreted protein which directly binds to LRP5/6 and has been shown to be the inhibitor of wnt/β-catenin signaling^11^,^12^. Elevated expression of Dkk-1 may thus play as a negative regulator for modulating wnt/β-catenin signal pathway.

The self-renewal and differentiation of hematopoietic stem cells (HSCs) are modulated by their surrounding niche in bone marrow (BM), which is composed of several types of BM stromal cells including mesenchymal stem/stromal cells (MSCs)^13^,^14^,^15^,^16^. Within the normal and leukemic BM microenvironment, BM-MSCs interact with HSCs or leukemic stem cells (LSCs) and produce pro-inflammatory cytokines/chemokines in a paracrine manner to regulate signaling that support cell survival and self-renewal^15^,^16^,^17^,^18^,^19^. It has been reported that secreted factors by LSCs or direct contact of LSCs with MSCs leads to educating MSCs or other stromal cells, thereby modulating or hijacking the homeostasis in the BM niche^17^,^18^,^19^. It has also been demonstrated that human MSCs inhibit K562 cell proliferation by secreting Dkk-1^10^,^20^. LSCs therefore interplay and synergize with educated BM-MSCs or surrounding stromal cells to promote leukemia progression as well as resistance to chemotherapy.

Interactions between leukemia-stromal cells by VLA-4/fibronectin or VLA-4/VCAM-1 are associated with the overall survival of AML patients^21^ and NFκB-mediated chemoresistance^22^. The VCAM-1/VLA-4 axis triggers NFκB activation and its consequent signaling which regulates cross-talk between leukemia and stromal cells^22^. In addition, BM derived MSCs preserve from elimination LSCs of CML when treated by a tyrosine kinase inhibitor through induction of N-cadherin and activation of wnt/β-catenin^23^. These results indicate that direct interaction of leukemia or LSCs with MSCs may contribute to enhanced survival and engraftment of LSCs as well as drug resistance in the BM.

LSCs respond to a number of supportive factors secreted by stromal cells in the BM microenvironment, including IL-6, IL-8 and stromal cell-derived factor-1 (SDF-1 or CXCL12)^24^,^25^. Among the secreted stromal factors, IL-6, IL-8 and are proinflammatory cytokines released by leukemia-associated stromal cells and are involved in maintaining the leukemic microenvironment for LSCs^24^,^25^,^26^,^27^,^28^. It has been shown that IL-6 controls leukemic progenitor cell fate and contributes to CML progression^29^. In addition, IL-8 released from BM-derived MSCs act as a mediator in the leukemic environment^26^,^27^. The IL-8-CXCR2 axis is overexpressed in LSCs and their expression is associated with a worse prognosis of AML and myelodysplastic syndromes patients (MDS)^28^. The IL-8/CXCR2 pathway expressed in leukemia-stromal cells is considered to be a potential prognostic factor and therapeutic target for leukemia and MDS^26^.

Dong-Chong-Xia-Cao is one of the commonly used traditional medicine which comprises the fungus (*Cordyceps sinesis*) and its infected larvae (*Hepialus armoricamus*)^30^,^31^. Cordycepin, also known as 3-deoxyadenosine, is a major active ingredient extracted from Dong-Chong-Xia-Cao and has been suggested to possess anti-leukemia properties^32^,^33^,^34^,^35^,^36^,^37^. However, cordycepin quickly loses its activity in animal models due to its short half-life^38^. As an adenosine analog, cordycepin is converted into inosine by adenosine deaminase (ADA)^39^. Co-administration of cordycepin with an ADA inhibitor (ADAi) or increasing cordycepin stability is considered a useful approach for leukemia therapy^40^,^41^,^42^,^43^,^44^. Cordycepin was reported to impair wnt/β-catenin signaling by inducing degradation of β-catenin in leukemia^35^. However, whether cordycepin suppresses leukemia progression via paracrine regulation has never been investigated. Our aim was to study the mechanism of how cordycepin contributes to suppressing leukemia’s progression. Our results suggest that cordycepin is a potential agent or adjuvant for leukemia therapy by targeting LSCs and disrupting the leukemia-MSC association.

## Results

### Cordycepin reduces the ratio of LSCs and induces Dkk-1 expression via autocrine and paracrine regulation

**Figure 1:**
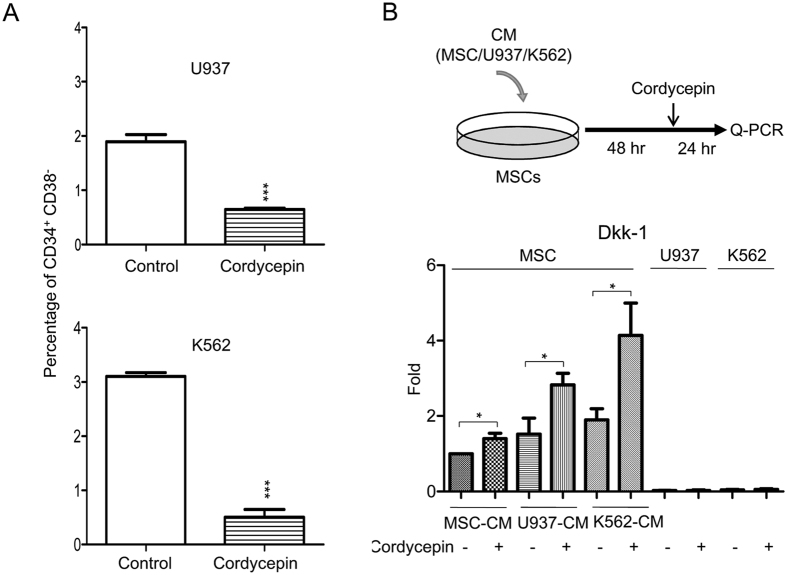
Cordycepin reduces the ratio of LSCs and induces Dkk-1 expression via autocrine and paracrine regulation. (**A**) U937 and K562 cells were treated with 50 μM cordycepin for 24 h. Percentages of CD34^+^CD38^−^ cells were determined by flow cytometry analysis. These results are from three independent experiments. Scale bars: mean ± SEM. *P < 0.05, ***P < 0.001. (**B**) Upper panel, a flow chart of the experimental design: MSCs were incubated with MSC-CM, U937-CM and K562-CM for 48 h followed by 50 μM cordycepin for an additional 24 h. Expression of Dkk-1 in (lower panel) MSCs, U937 and K562 was determined by Q-PCR (N = 5). Scale bars: mean ± SEM. *P < 0.05.

The population of CD34^+^CD38^−^ cells is considered to be LSC or leukemia initiating cells^45^,^46^. To investigate whether cordycepin influences the stemness of myeloid leukemia, U937 and K562 cells were treated with cordycepin (50 μM) for 24 h and the percentage of CD34^+^CD38^−^ cells were determined by flow cytometry analysis. Cordycepin significantly reduced the percentage of CD34^+^CD38^−^ cells in both U937 (Fig. 1A, upper panel) and K562 (Fig. 1A, lower panel).

We postulated that MSCs are educated by leukemia. We therefore examined whether cordycepin influences β-catenin signaling of leukemia in a paracrine manner. MSCs were pretreated with conditioned media of MSCs (MSC-CM), U937 (U937-CM) and K562 (K562-CM) for 48 h, followed by cordycepin treatment for additional 24 h (procedure illustrated in Fig. 1B, upper panel). Expression of Dkk1, an antagonist of wnt/β-catenin signaling in leukemia and MSCs was determined by quantitative real-time PCR (Q-PCR). Cordycepin significantly induced Dkk1 expression in MSC-CM, U937-CM, and K562-CM treated MSCs, whereas cordycepin had no effect on Dkk1 expression in U937 and K562 cells (Fig. 1B, lower panel). These results indicate that cordycepin may attenuate wnt/β-catenin signaling in leukemia cells by both autocrine and paracrine regulation.

### Cordycepin suppresses leukemia’s attachment with MSCs and reduces expression of N-cadherin in leukemia and VCAM-1 in leukemic CM-treated MSCs

**Figure 2:**
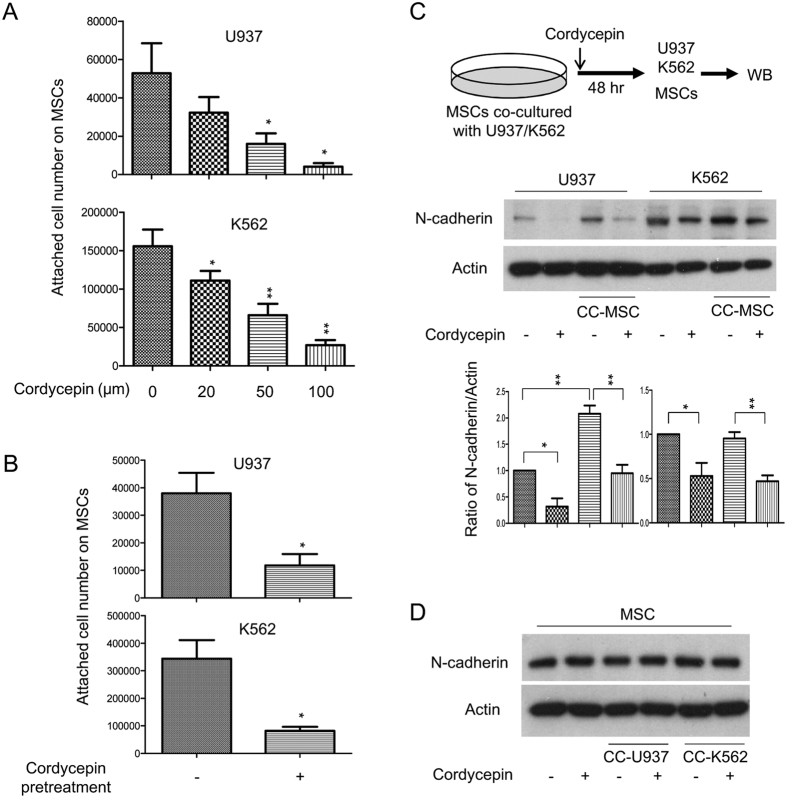
Cordycepin suppresses leukemia attachment with MSCs and reduces expression of N-cadherin in MSC-co-cultured leukemia. (**A**) U937 and K562 cells were plated on MSCs containing different concentrations of cordycepin (0, 20, 50 and 100 μM) for 24 h. (**B**) U937 and K562 cells were pre-treated with cordycepin (50 μM) for 24 h and subsequently plated onto MSCs. The number of attached live cells was examined by trypan blue staining. These results are from four independent experiments. Scale bars: mean ± SEM. *P < 0.05; **P < 0.01. (**C**) Upper panel, a flow chart of the experimental design: U937 and K562 cells were co-cultured with MSCs for 48 h. Expression of N-cadherin in U937 and K562 (middle panel) was determined by Western blot analysis and quantified by densitometry (lower panel). This blot is a representative figure from three independent experiments and the quantification is indicated as mean ± SEM. *P < 0.05; **P < 0.01. (**D**) MSCs was determined by Western blot analysis. Actin was used as loading control. These figures are cropped blots and the full-length blots are presented in Supplementary Fig. 6A,B.

To elucidate whether cordycepin affects association of leukemia with niche cells, we examined whether cordycepin influences leukemia’s attachment to MSCs. U937 and K562 cells were plated on MSCs and consequently treated with different doses of cordycepin. Cordycepin significantly suppressed leukemia cell attachment to MSCs in a dose-dependent manner (Fig. 2A). To further confirm the suppressive effect of cordycepin on leukemia’s attachment with MSCs, U937 and K562 cells were pre-treated with cordycepin (50 μM) for 24 h and subsequently plated on the MSCs for additional 24 h. Cordycepin significantly reduced U937 and K562 attached on MSCs (Fig. 2B). An earlier study has demonstrated that the leukemia-microenvironment association protects CML stem/progenitor cells from tyrosine kinase inhibitor treatment through N-cadherin signaling^23^. We therefore examined whether cordycepin modulates N-cadherin expression in leukemia and MSCs. U937 and K562 cells were co-cultured with MSCs and treated with or without 50 μM cordycepin for 48 h. U937 or K562 cells were removed from MSCs and N-cadherin expression was determined by Western blot analysis (illustrated in Fig. 2C, upper panel). Co-culturing of U937 with MSCs induced but co-culture of K562 with MSCs had no significant effect of N-cadherin expression, whereas cordycepin markedly suppressed N-cadherin in U937 and K562 cells (Fig. 2C, lower panel). In contrast, neither co-culturing with leukemia nor cordycepin treatment influenced N-cadherin expression in MSCs (Fig. 2D).

**Figure 3:**
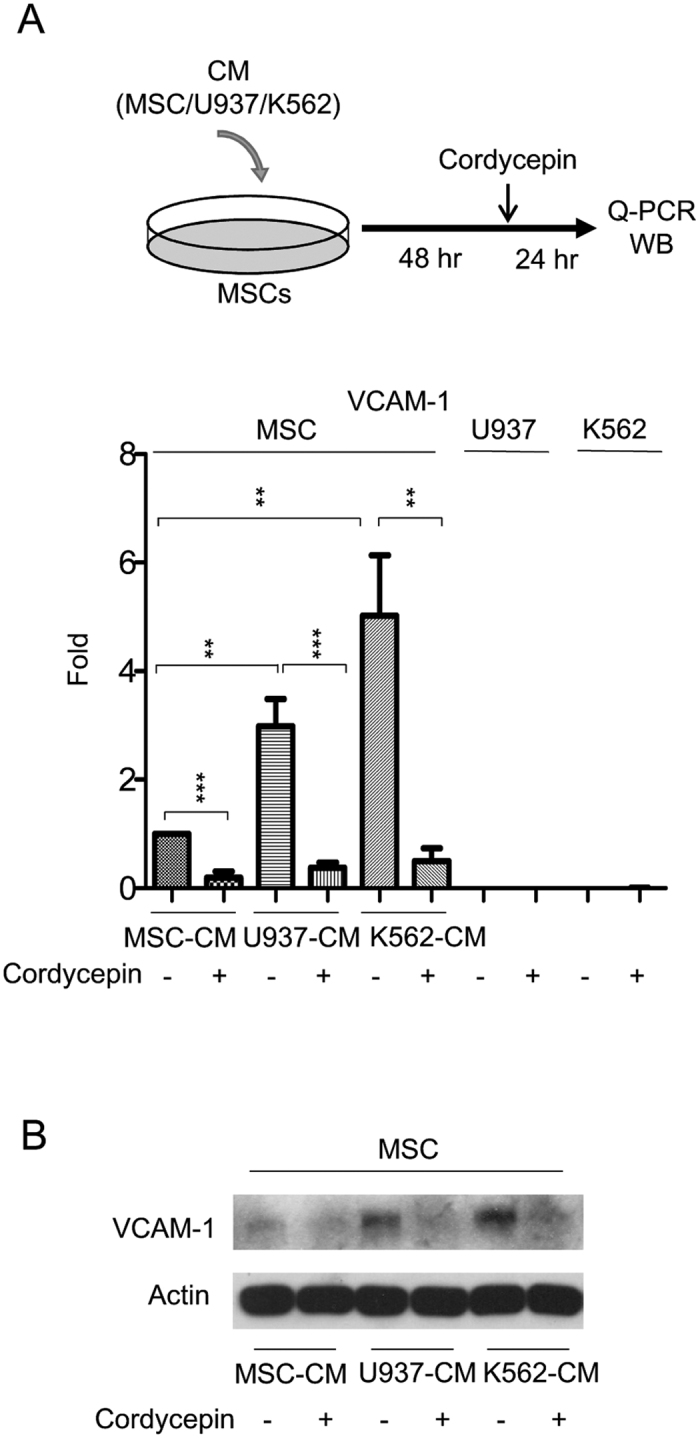
Cordycepin suppresses VCAM-1 in leukemic CM-treated MSCs. (**A**) Upper panel, a flow chart of the experimental design: MSCs were incubated with MSC-CM, U937-CM and K562-CM for 48 h followed by 50 μM cordycepin for an additional 24 h. Expression of VCAM-1 in (lower panel) MSCs, U937 and K562 was determined by Q-PCR (N = 5). Scale bars: mean ± SEM. **P < 0.01; ***P < 0.001, and (**B**) Western blot analysis. Actin was used as loading control. This figure is a cropped blot and the full-length blot is presented in Supplementary Fig. 7.

A recent study has indicated that the leukemia-stroma VCAM-1/VLA-4 interaction is involved in chemoresistance of leukemia^22^. We examined whether cordycepin regulates VCAM-1/VLA-4 expression in leukemia-stromal cells. MSCs were incubated with MSC-CM, U937-CM and K562-CM for 48 h, followed by cordycepin treatment for an additional 24 h. MSCs and leukemia cells were harvested and expression of VCAM-1 was examined by Q-PCR and Western blotting (illustrated in Fig. 3A, upper panel). U937-CM and K562-CM induced VCAM-1 in MSCs (Fig. 3A, lower panel). These results were further confirmed by Western blot analysis (Fig. 3B). Intriguingly, cordycepin significantly reduced VCAM-1 expression in leukemic CM-treated MSCs but had no effect in leukemia cells (Fig. 3A, lower panel). We further determined VLA-4 expression by Q-PCR of integrin β1 in U937 and K562 cells. Only a slight expression level change was induced by cordycepin treatment (Supplementary Fig. 1).

### Cordycepin abolishes leukemic CM-induced IL-8 and IL-6 in MSCs

**Figure 4:**
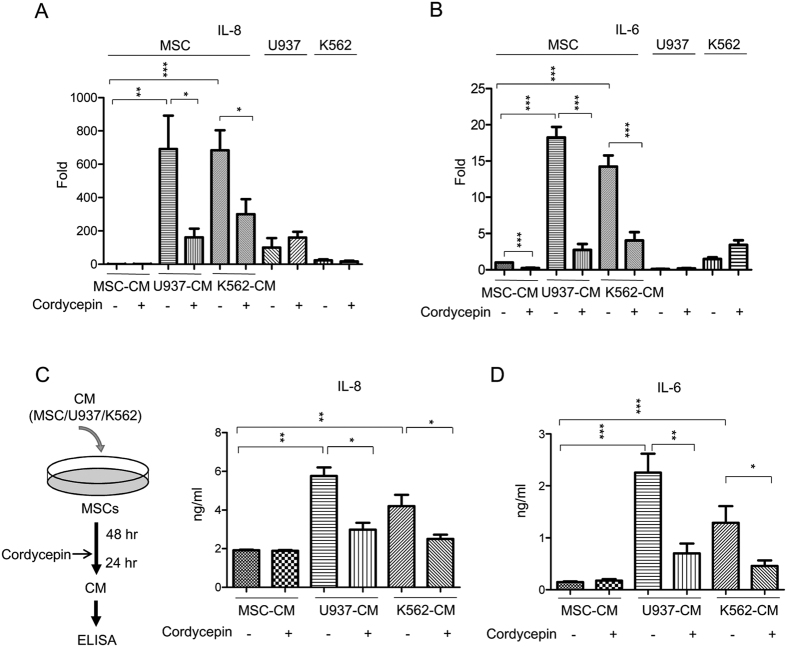
Cordycepin abolishes leukemic CM-induced IL-8 and IL-6 in MSCs. MSCs were incubated with MSC-CM, U937-CM and K562-CM for 48 h followed by 50 μM cordycepin for an additional 24 h. Expression of (**A**) IL-8 or (**B**) IL-6 in MSCs, U937 and K562 was determined by Q-PCR. (**C**) Left panel, a flow chart of the experimental design: MSCs were incubated with MSC-CM, U937-CM or K562-CM for 48 h followed by 50 μM cordycepin for an additional 24 h. CM were harvested and level of (Right panel) IL-8 (N = 4) and (**D**) IL-6 (N = 5) were determined by ELISA assay. Scale bars: mean ± SEM. *P < 0.05; **P < 0.01; ***P < 0.001.

IL-8 and IL-6 are pro-inflammatory cytokines involved in modulating the progression of myelogenous leukemia^24^,^25^,^26^,^27^,^28^. We examined whether MSCs are a source of stromal cell IL-8 and IL-6 secretion. We found that U937-CM and K562-CM abundantly induced IL-8 and IL-6 expression in leukemic CM-treated MSCs (Fig. 4A,B). We next incubated U937 and K562 cells in CM that was harvested from leukemic CM-incubated MSCs (illustrated in Supplementary Fig. 2A). CM from leukemic CM-treated MSCs promoted U937 and K562 cell proliferation (Supplementary Fig. 2B). Additionally, we found that cordycepin suppressed IL-8 and IL-6 expression in leukemic CM-treated MSCs (Fig. 4A,B). In contrast, cordycepin did not affect IL-8 and IL-6 expression in U937 and K562 cells (Fig. 4A,B). We further harvested CM from leukemic CM-treated MSCs and determined IL-8 and IL-6 levels by ELISA (procedure illustrated in Fig. 4C, left panel). Cordycepin significantly abrogated leukemic CM-induced IL-8 and IL-6 (Fig. 4C, right panel and 4D). As the IL-8/CXCR2 axis has been implicated as a therapeutic target against MDS and AML^28^, we examined CXCR2 expression by Q-PCR analysis in U937 and K562 cells which were co-cultured with MSCs. Neither the co-culture nor cordycepin significantly influenced CXCR2 expression which were analyzed by Q-PCR and flow cytometry (Supplementary Fig. 3A–D).

### The role of β-catenin in leukemia hijacks MSCs

**Figure 5:**
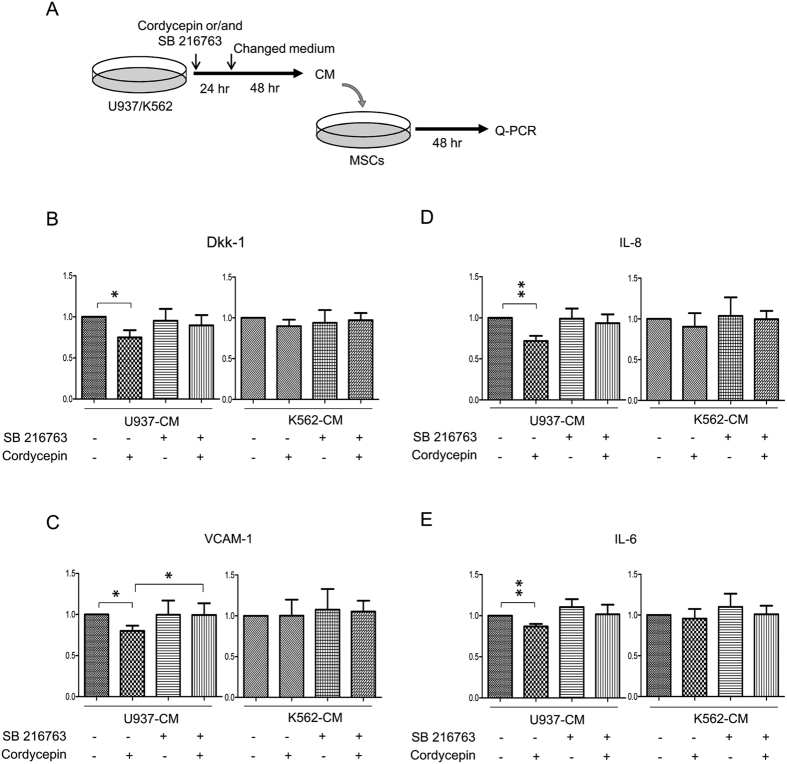
The role of β-catenin in leukemia hijacks MSCs. (**A**) Upper panel, a flow chart of the experimental design: U937 and K562 cells were treated with 50 μM cordycepin or/and 10 μM SB216763 for 24 h followed by change of medium for an additional 48 h. CM was removed and added to MSCs for a further 48 h. Expression of (**B**) Dkk-1, (**C**) VCAM-1, (**D**) IL-8 and (**E**) IL-6 in MSCs were determined by Q-PCR (N = 4). Scale bars: mean ± SEM. *P < 0.05; **P < 0.01.

An earlier study has indicated that cordycepin induces β-catenin degradation through activating GSK-3β and that a pharmacological inhibitor of GSK-3β significantly restored cordycepin-reduced β-catenin^35^. As β-catenin is a crucial factor for maintaining the stemness of LSCs, we investigate whether β-catenin is involved in regulating the leukemia-stromal association. U937 and K562 cells were treated with cordycepin or/and with the GSK-3β inhibitor (SB 216763) for 24 h. U937-CM and K562-CM were harvested and were added into MSCs for 48 h followed by Q-PCR analysis (procedure illustrated in Fig. 5A). CM derived from cordycepin or/and SB 216763-treated K562 cells did not affect expression of Dkk-1, VCAM-1, IL-8 and IL-6 in MSCs (Fig. 5B–E, right panels) whereas CM derived from cordycepin-treated U937 slightly suppressed expression of Dkk-1, VCAM-1, IL-8 and IL-6 in MSCs (Fig. 5B–E, left panels). Moreover, cordycepin-suppressed VCAM-1 can be restored by treatment of SB 216763 (Fig. 5C, left panels).

### Cordycepin suppresses VCAM-1 and IL-8 expression through attenuation of NFκB activation in leukemic CM-treated MSCs

**Figure 6:**
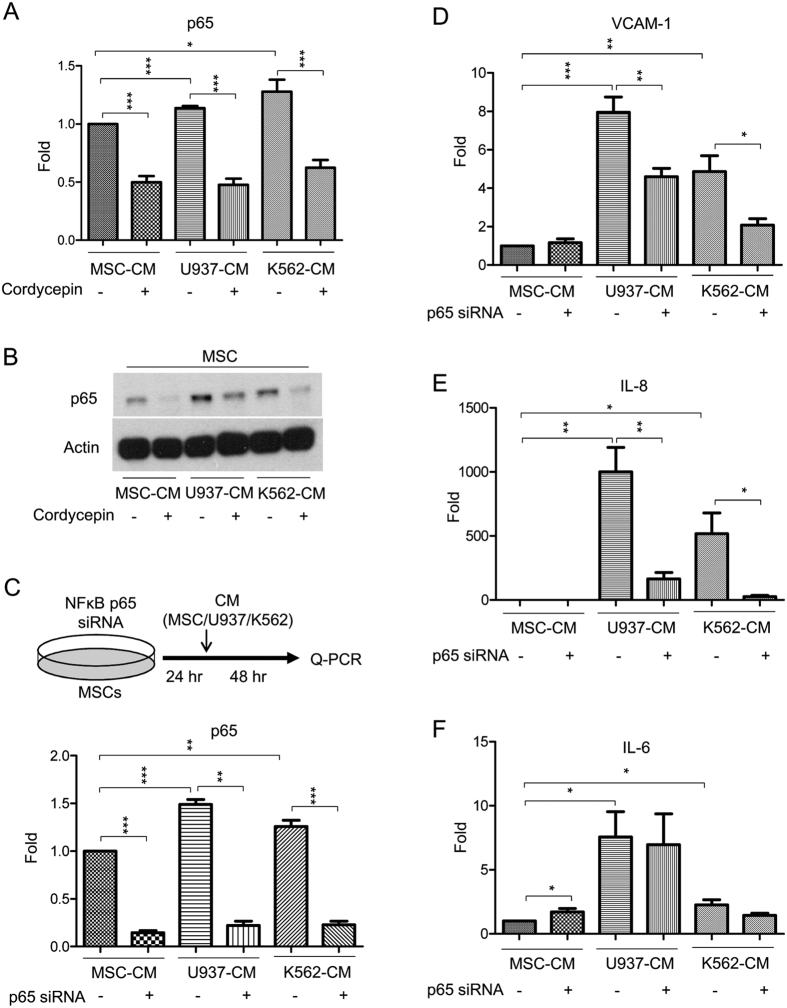
Cordycepin suppresses VCAM-1 and IL-8 expression through attenuation of NFκB activation in leukemic CM-treated MSCs. (**A**) MSCs were incubated with MSC-CM, U937-CM and K562-CM for 48 h followed by 50 μM cordycepin for an additional 24 h. Expression of p65 was determined by Q-PCR (N = 5) and (**B**) Western blot analysis. This figure is a cropped blot and the full-length blot is presented in Supplementary Fig. 8. (**C**) Upper panel, a flow chart of the experimental design: MSCs were transfected with p65 siRNA for 24 h followed by incubation with MSC-CM, U937-CM and K562-CM for an additional 48 h. Expression of (lower panel) p65, (**D**) VCAM-1, (**E**) IL-8 and (**F**) IL-6 was determined by Q-PCR (N = 4). Scale bars: mean ± SEM. *P < 0.05; **P < 0.01; ***P < 0.001. Actin was used as loading control.

NFκB has been implicated in regulating the leukemia-stromal interaction^47^,^48^,^49^. We decided to examine whether NFκB is involved in cordycepin’s disruption of the leukemia-MSC association. We found that U937-CM and K562-CM induced expression of NFκB’s subunit p65 in leukemic CM-incubated MSCs whereas cordycepin abolished leukemic CM-induced p65 expression (Q-PCR analysis in Fig. 6A and Western blot analysis in Fig. 6B). We further performed a knockdown of p65 by transfection with p65 siRNA. MSCs were transfected with p65 siRNA for 24 h, followed by the addition of leukemic and control CM (experimental procedure illustrated in Fig. 6C, upper panel). Reduced expression of p65 by siRNA was confirmed by Q-PCR analysis (Fig. 6C, lower panel). Silencing p65 attenuated leukemic CM-induced VCAM-1 (Fig. 6D) and IL-8 (Fig. 6E) expression but had no significant effect on IL-6 (Fig. 6F). These results suggest that leukemic CM induced VCAM-1 and IL-8 expression is through NFκB activation in MSCs. Cordycepin suppressed NFκB expression, thereby inhibiting VCAM-1 and IL-8 expression in MSCs.

### Cordycepin prolongs survival of U937 and K562-inoculated mice

To investigate whether cordycepin suppresses leukemia progression, we performed *in vivo* experiments with U937 and K562- inoculated NOD-SCID mice. U937 and K562 cells were pre-treated with or without cordycepin for 24 h and were consequently inoculated into NOD-SCID mice by intravenous injection. Pretreatment of cordycepin prolonged the survival rate in U937 and K562-inoculated mice (Supplementary Fig. 4A,B).

**Figure 7:**
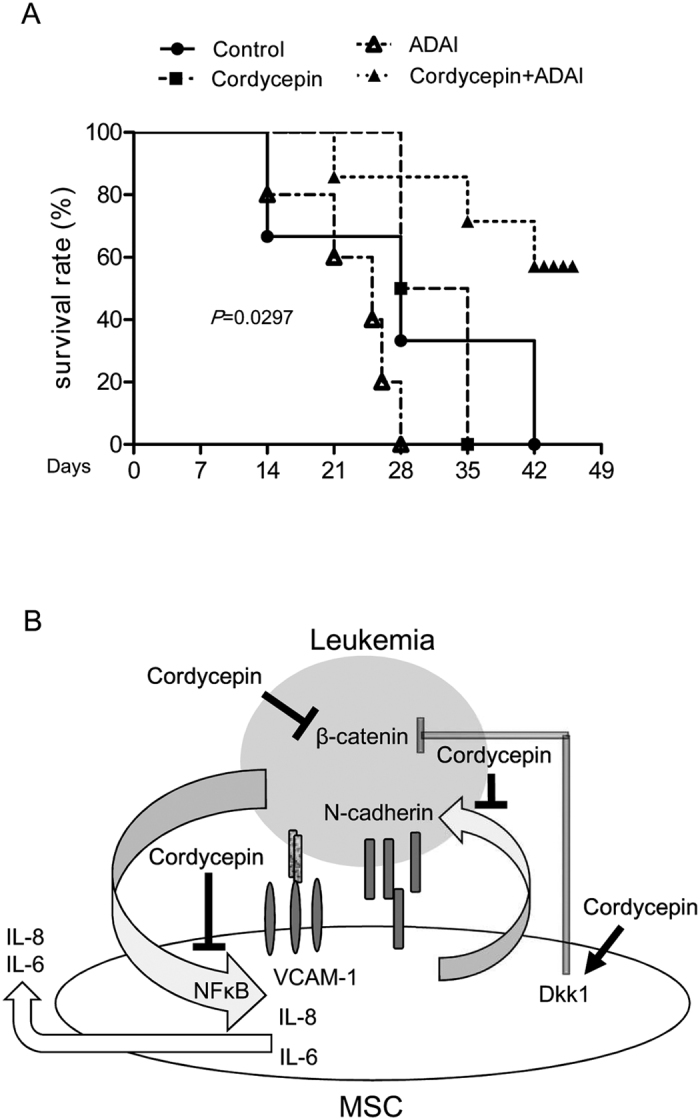
Cordycepin prolongs survival of U937-inoculated mice. (**A**) U937 cells were intravenously injected into NOD-SCID mice. After one week, 5 mg/kg cordycepin and/or 1 mg/kg pentostatin (ADAi) was intraperitoneal injected and the survival of mice was demonstrated. (**B**) An illustration scheme for the actions of cordycepin in eliminating stemness and disrupting association with mesenchymal stromal cells.

Cordycepin has a short half-life *in vivo* due to degradation by adenosine deaminase (ADA)^39^. To examine the *in vivo* effect of cordycepin on leukemia suppression, we administered U937 cells intravenously to NOD-SCID mice. U937-inoculated mice were intraperitoneally injected with cordycepin with or without pentostatin, an ADA inhibitor (ADAi). Cordycepin combined with ADAi significantly prolonged the survival rate of mice whereas cordycepin or pentostatin alone did not (Fig. 7A).

## Discussion

Targeting the association of cancer stem cells with their surrounding niche is a potential therapeutic approach or adjuvant treatment for malignancies, particularly drug-resistant tumors. Bone marrow microenvironment hijacking by LSCs has been implicated in leukemia development and chemoresistance^17^,^18^,^19^. Wnt/β-catenin signaling is crucial for LSC development^1^,^2^,^3^,^4^,^5^,^6^,^7^,^8^,^9^ and paracrine Dkk1 secreted from MSCs inhibits leukemia and cancer cell proliferation^10^,^20^. An earlier study has indicated that cordycepin suppresses β-catenin and cell proliferation in U937, K562 and THP-1 cells^35^. In this study, we found U937-CM and K562-CM did not reduce Dkk-1 expression in MSCs (Fig. 1B). However, cordycepin induced Dkk1 expression in MSC-CM, U937-CM and K562-CM treated MSCs (Fig. 1B). These results suggest that Dkk-1 expression can be induced by cordycepin in either normal or leukemia-CM treated MSCs. Cordycepin has autocrine and paracrine anti-leukemia effects via attenuating wnt/β-catenin signaling in leukemia and associated stromal cells.

Cellular N-cadherin and VCAM-1 are involved in the leukemia-stroma interaction and protect leukemia cells from tyrosine kinase inhibitors or chemotherapy^22^,^23^. Our findings indicate that co-culturing leukemia with MSCs induces N-cadherin expression in U937 cells (Fig. 2C) and the incubation of MSCs with leukemic-CM (U937-CM and K562-CM) enhances VCAM-1 expression in MSCs (Fig. 3A,B). Expressions of N-cadherin in leukemia cells and VCAM-1 in MSCs were significantly impaired by cordycepin treatment (Figs 2C,3A and B). Moreover, cordycepin has been reported to possess suppressive effect on cell proliferation. In this study, we found that cordycepin reduced attached cell number of U937 and K562 on MSCs. This effect may due to suppress N-cadherin, VCAM-1 or other adhesive molecules in leukemia and MSCs. However, it may be possible caused by reduced cell proliferation or viability. In addition, IL-6, IL-8 and several other pro-inflammatory cytokines have been shown to mediate the leukemia microenvironment in myeloid leukemia^24^,^25^,^26^,^27^,^28^. We found an induction effect of IL-6 and IL-8 by paracrine regulation in U937-CM and K562-CM treated MSCs which was significantly abrogated by cordycepin (Fig. 4A–D). In this study, we show for the first time that cordycepin disrupts leukemia-MSC association through downregulation of N-cadherin in leukemia cells and attenuation of leukemia-CM-induced VCAM-1, IL-6 and IL-8 in MSCs. Taken together, cordycepin is a potential anti-leukemia agent that disrupts the leukemia-MSC association and eliminates leukemia stem cell activity (Fig. 7B).

Cordycepin suppresses β-catenin via GSK-3β signaling activation and treatment with a pharmacological inhibitor of GSK-3β restored cordycepin-reduced β-catenin in leukemia^35^. We then examined whether leukemic GSK-3β/β-catenin signaling involves the paracrine regulation for VCAM-1, IL-6 and IL-8 expression in MSCs. CM harvested from U937 and K562 cells pre-treated with cordycepin or/and SB 216763 has no significant or only partial effect on Dkk-1, VCAM-1, IL-6 and IL-8 expression in MSCs (Fig. 5B–D). These results suggest that β-catenin signaling in leukemia is not a major factor in the paracrine regulation of the leukemia niche. Previous studies have implicated leukemia-secreted factors or exosomes in regulating crosstalk between leukemia and bone marrow stromal cells^26^,^27^. We found that leukemia-CM may educate MSCs thereby inducing VCAM-1, IL-6, IL-8 and other effectors on promoting leukemia progression. Cordycepin has a profound effect on attenuating the expression of N-cadherin in leukemia and VCAM-1, IL-8 and Il-6 in MSCs. However, the detailed mechanism and the involved secreted factors in hijacking the leukemia niche needs further investigation.

NFκB was shown to regulate tumor progression via a paracrine regulation in the tumor microenvironment^47^,^48^,^49^. Focal adhesion kinase (FAK) is overexpressed in AML and associates with poor prognosis^50^. It has been reported that FAK-expressing AML regulates IL-6, IL-8, CXCL-12 and Dkk1 expression in MSCs thereby hijacking the leukemic niche^25^ and an earlier study indicated that cordycepin downregulates integrin/FAK expression and cell migration via impairing NFκB signaling in hepatocellular carcinoma^51^. We postulated that cordycepin affects FAK expression, cell adhesion molecules and pro-inflammatory cytokines through inactivating NFκB signaling. We found that U937-CM and K562-CM had no significant effect on FAK and CXCL-12 expression in MSCs (Supplementary Fig. 5A,B) when compared to VCAM-1, IL-8 or IL6. Intriguingly, cordycepin suppressed U937-CM and K562-CM-induced expression of NFκB (Fig. 6A,B), IL-8 (Fig. 4A), IL-6 (Fig. 4B), FAK (Supplementary Fig. 5A) and CXCL12 (Supplementary Fig. 5B) in MSCs. In addition, silencing of NFκB by siRNA significantly downregulated VCAM-1, IL-8 and IL-6 expression in MSCs (Fig. 6D–F). These results suggest that cordycepin may suppress expression of VCAM-1, IL-8, IL-6 as well as other related factors and interrupts the leukemia/MSC association through NFκB inactivation.

Cordycepin is the major ingredient of the caterpillar fungus Dong-Chong-Xia-Cao and is an analogue of adenosine. Cordycepin has a short *in vivo* half-life in mice due to ADA degradation^38^,^39^. We injected cordycepin and/or ADAi into U937- inoculated NOD-SCID mice. Injection of cordycepin or ADAi did not extent mice survival whereas a combination of cordycepin and ADAi significantly prolonged the survival rate of U937-inoculated mice (Fig. 7A). These results echo the suggestion found in earlier studies that stability issue of cordycepin should be considered in future clinical applications^38^,^39^,^40^,^41^,^42^,^43^,^44^. Cordycepin combined with ADAi or developing stable cordycepin derivative compounds is therefore a potential adjuvant used for leukemia therapy.

## Methods

### Cell culture and preparation of CMs

Human MSCs were isolated from the bone marrow of normal donors described previously^52^,^53^ after signing informed consent and all experiments and protocols were performed in accordance with guidelines and regulations approved according to the procedures of the Institutional Review Board of National Taiwan University Hospital (NTUH), Taiwan. MSCs were maintained and cultured in low-glucose Dulbecco’s modified Eagle’s medium (DMEM, Gibco) supplemented with 10% fetal bovine serum (Gibco) and penicillin-streptomycin (100 U/ml, 100 μg/ml; Gibco). The U937 and K562 cells were cultured in Roswell Park Memorial Institute (RPMI) medium (Gibco) supplemented with 10% FBS and penicillin-streptomycin. The U937-CM and K562-CM were harvested from medium incubated with 5 × 10^4^ cell/ml for 48 h followed by centrifugation at 1500 rpm for 5 min. CMs were stored at −80 °C.

### Co-culture and cell attachment of leukemia with MSCs

Adherent bone marrow MSCs were seeded in 12 well plates to reach 80–90% confluency. 2 × 10^5^ of U937 or K562 cells were cultured onto MSCs supplemented with or without 50 μM cordycepin (Sigma) for 24 h. The suspended cells were removed and the contacted cells were gently washed away from MSCs by pipetting. The viability of the leukemia cells were counted using trypan blue staining.

### Flow cytometry analysis

FITC-labeled anti-human CD34, PE-labeled anti-human CD38 and PE-labeled anti-human CXCR2 (eBiosciences) were used to determine the population of CD34^+^/CD38^−^ and CXCR2 positive U937 and K562 cells. U937 and K562 cells were treated with 50 μM cordycepin for 24 hours followed by incubation on ice with an Fc receptor binding inhibitor (eBiosciences) for 15 min. Cells were incubated for an additional 60 min at 4 °C with the antibodies against CD34, CD38 or CXCR2 in the dark. The cell were washed with PBS three times then fixed with fixation buffer (Biolegend). Flow cytometric acquisition was performed using an FACS Calibur (BD Biosciences) and data were analyzed by CellQuest software (BD Biosciences).

### Western blot analysis

Cells were lysed with RIPA (Millipore) buffer containing cocktail protease inhibitors (Roche). The lysates were centrifuged at 15,000 rpm at 4 °C for 10 min. The supernatant was collected and total protein was determined using a Bio-Rad protein assay kit (Bio-Rad Laboratories). Equal amounts of total protein from each sample were loaded into the gradient SDS-PAGE gel, and the proteins were then transferred onto PVDF membranes. The membranes were blocked with 5% non-fat milk and probed with specific antibodies against N-cadherin (BD), NFκB p65 (Santa Cruz), V-CAM1 (Santa Cruz), β-catenin (BD) and β-Actin (sigma). The membranes were immersed in horseradish peroxidase-conjugated secondary antibody and immunoreactive bands were exposed on an X-ray film.

### Real-Time Quantitative PCR (Q-PCR)

Total RNA was isolated by RNAzol^®^ RT (Molecular Research Center) and cDNA was synthesized using an ABI RT Kit (ABI Applied Biosystems). RNA expression levels were analyzed using quantitative real-time PCR (Q-PCR) with specific primers (Supplementary Table 1). PCR reactions were performed using SYBR Green (Kapa Biosystems) in an ABI Prism 7900 System (ABI Applied Biosystems). The quantity of the specific genes was normalized with GAPDH of the same sample. Fold change was determined as the ratio of the each control sample. All PCR reactions were performed in triplicate.

### ELISA

Levels of IL-6 and IL-8 in medium were measured using ELISA kits purchased from eBioscience (San Diego, CA) according to the manufacturer’s instructions. The capture antibody was coated on the ELISA plates and incubated overnight at 4 °C. After washing with PBS containing 0.1% Tween-20 (PBST), the plates were treated with a blocking buffer at room temperature for 1 h. Diluted samples or standards of IL-6 or IL-8 were added and incubated overnight at 4 °C. The detection antibody was incubated for 1 h and Avidin-HRP was incubated for 30 min at room temperature. Absorbance was measured at 450 nm.

### Transfection of siRNA

MSCs were seeded in 6 well plates the day before transfection. Transfection was performed using Lipofectamine^®^ RNAiMAX Reagent (Invitrogen). 15 pmole of scramble or siRNA for human NFκB p65 (Sequences: GCCCUAUCCCUUUACGUC and GACGUAAAGGGAUAGGGC, Santa Cruz) was added into the transfection mixture. Transfection efficiency was determined by Q-PCR.

### U937-inoculated SCID mice model

The SCID mice as a model for U937 inoculation was previously established^54^. The protocol of this study for U937-inoculated SCID mice was performed in accordance with the guidelines and regulations approved by the Institutional Animal Care and Use Committee of the National Health Research Institutes. NOD-SCID mice (8 weeks old) were purchased from the National Laboratory Animal Center and housed in microisolator cages at a specific pathogen-free facility at the Laboratory Animal Center, National Health Research Institutes of Taiwan. NOD-SCID mice received of 300 cGy X-ray irradiation to eliminate their hematopoietic systems. After 24 h, 5 × 10^6^ U937 cells were intravenously injected into the tail vein. After one week, the mice were divided into four groups: control (N = 8), cordycepin (N = 6), ADAi (N = 5) and cordycepin combined with ADAi (N = 8). U937-inoculated mice were intraperitoneally injected with 5 mg/kg cordycepin and/or 1 mg/kg pentostatin (ADAi) (Santa Cruz) in the lower-right area of the abdominal cavity. The treatment was given once every two days for three times.

### Statistical analysis

Data are presented as mean ± SEM. Student’s t-test was used to analyze differences between 2 experimental groups. Mantel-COX test was used to analyze the survival of the mice. A *P*-value of less than 0.05 was considered statistically significant. *, ** and *** Indicate *P*-value < 0.05, 0.01, and 0.001, respectively.

## Additional Information

**How to cite this article**: Liang, S.-M. *et al*. Cordycepin disrupts leukemia association with mesenchymal stromal cells and eliminates leukemia stem cell activity. *Sci. Rep.* **7**, 43930; doi: 10.1038/srep43930 (2017).

**Publisher's note:** Springer Nature remains neutral with regard to jurisdictional claims in published maps and institutional affiliations.

## Supplementary Material

Supplementary Information
