# Supplementary material for: Cordycepin disrupts leukemia association with mesenchymal stromal cells and eliminates leukemia stem cell activity
Source: Sci Rep. 2017 Mar 7;7:43930. doi: 10.1038/srep43930 (PMC5339716; doi:10.1038/srep43930)
Supplement: Supplementary Information [file srep43930-s1.pdf]

Supplementary Information

Cordycepin disrupts leukemia association with mesenchymal stromal cells and eliminates leukemia stem cell activity

Shu-Man Liang, Yi-Jhu Lu, Bor-Sheng Ko, Yee-Jee Jan, Song-Kun Shyue, Shaw-Fang Yet & Jun-Yang Liou*

Supplementary Table 1. Oligonucleotide sequences for Q-PCR.

Name		Sequences (5' to 3')
GAPDH	F	CGC TCT CTG CTC CTC CTG TT
	R	CCA TGG TGT CTG AGC GAT GT
VCAM-1	F	TCC CTA CCA TTG AAG ATA CTG GAA A
	R	GCT GAC CAA GAC GGT TGT ATC TC
DKK-1	F	ACC CAG GCT CTG CAG TCA
	R	CCT GCA GGC GAG ACA GAT
IL-6	F	CCC CCA GGA GAA GAT TCC AAA G
	R	TTC TGC CAG TGC CTC TTT GCT G
IL8	F	AGC TGG CCG TGG CTC TCT
	R	CTG ACA TCT AAG TTC TTT AGC ACT CCT T
CXCR2	F	GCT CTG ACT ACC ACC CAA CCT TGA
	R	AGA AGA GCA GCT GTG ACC TGC TGT
p65	F	CCC CAC GAG CTT GTA GGA AAG
	R	CCA GGT TCT GGA AAC TGT GGA T
integrinα4	F	GCT TCT CAG ATC TGC TCG TG
	R	GTC ACT TCC AAC GAG GTT TG
integrinβ4	F	AGA CGA GAT GTT CAG GGA CC
	R	GGT CTC CTC TGT GAT TTG GAA
FAK	F	TCC CTA TGG TGA AGG AAG TC
	R	TTC TGT GCC ATC TCA ATC TC
CXCL12	F	ATG CCC ATG CCG ATT CTT
	R	GCC GGG CTA CAA TCT GAA GG

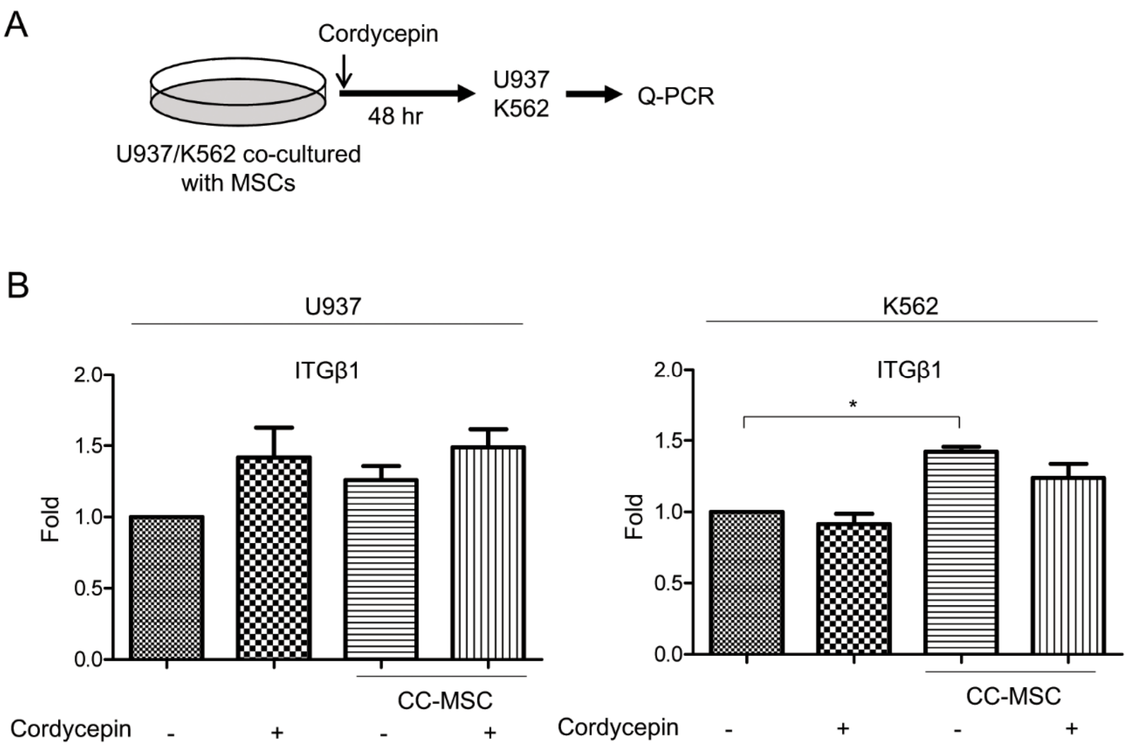

Supplementary Figure 1. Cordycepin has no effect on Integrinβ1 (ITGβ1) expression in leukemia co-cultured with MSCs. (**A**) A flow chart of the experimental design. (**B**) U937 and K562 cells were co-cultured with MSCs and treated with/without 50 μM cordycepin for 48 h. Expression of ITGβ1 in U937 (left panel) or K562 (right panel) was determined by Q-PCR (N=4). Scale bars: mean ± SEM. *, *P*<0.05.

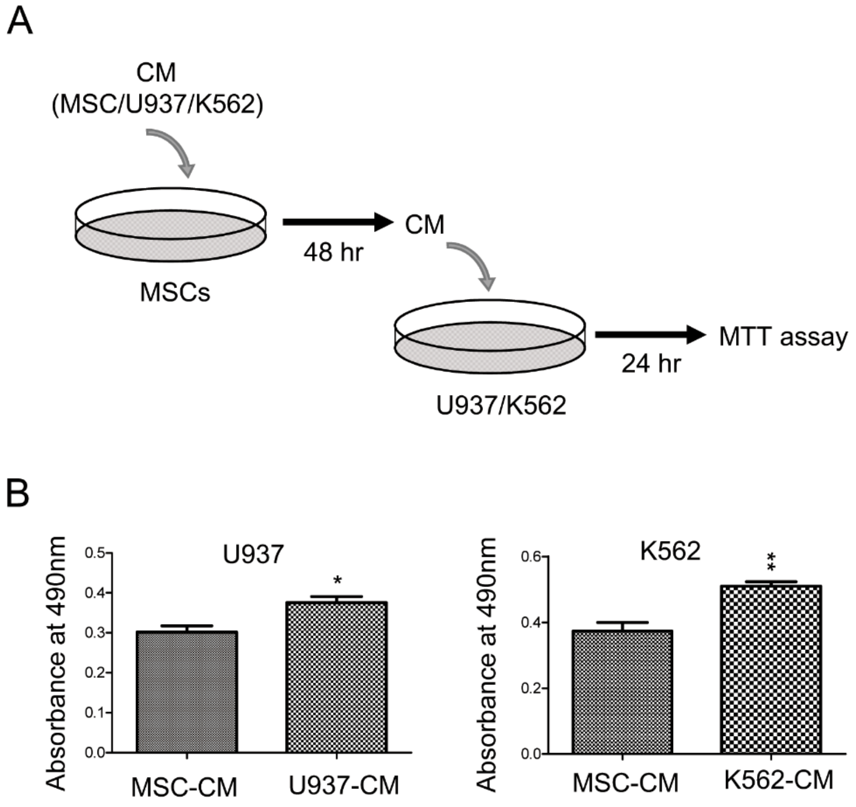

Supplementary Figure 2. CM from leukemic CM-treated MSCs induced U937 and K562 cell proliferation. (**A**) A flow chart of the experimental design. (**B**) U937 and K562 cells were incubated with CM harvested from MSCs which were pre-incubated with MSC-CM, U937-CM or K562-CM. Cell proliferation was analyzed by MTT assay (N=4). Scale bars: mean ± SEM. *, *P*<0.05; **, *P*<0.01.

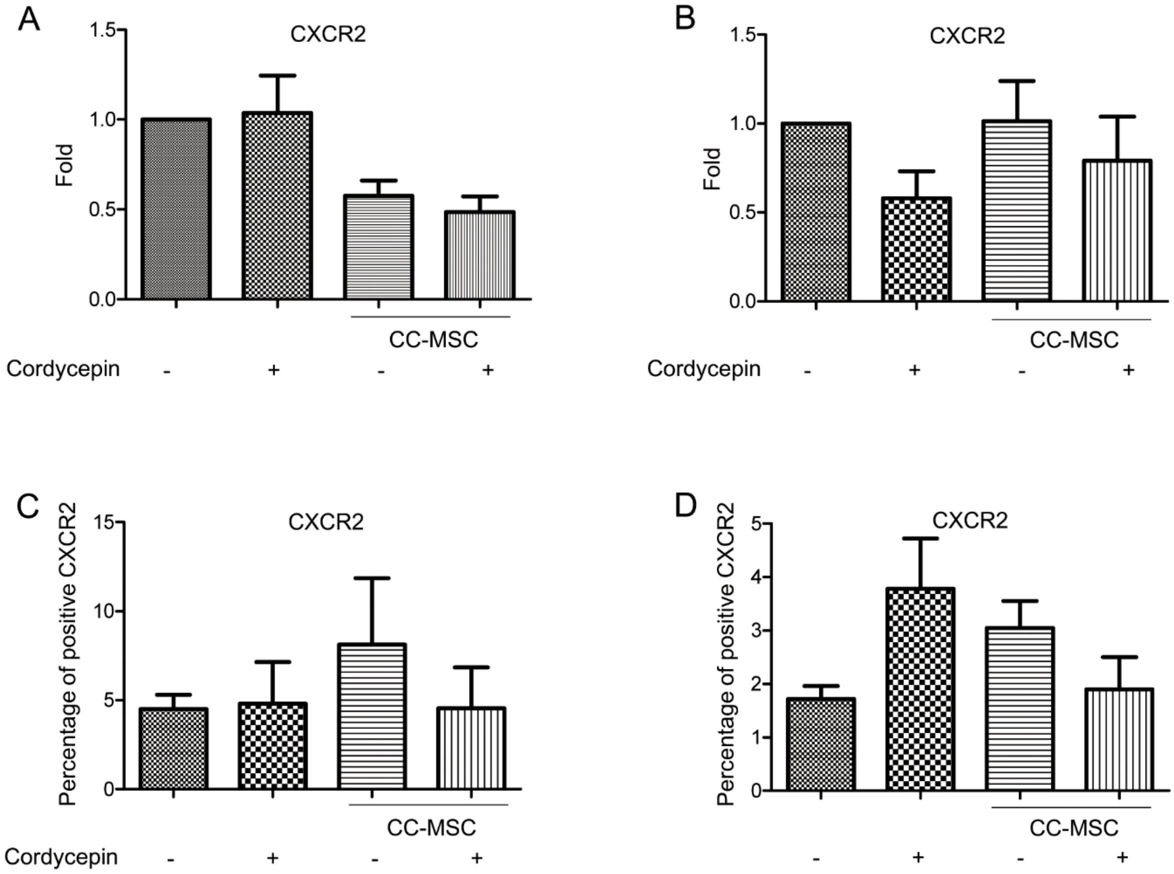

Supplementary Figure 3. Cordycepin has no effect on CXCR2 expression in leukemia co-cultured with MSCs. U937 and K562 cells were co-cultured with MSCs for 48 h followed by treated with/without 50 μM cordycepin for additional 24 h. Expression of CXCR2 in (A, C) U937 or (B, D) K562 (N=4) was determined by Q-PCR (A, B) and flow cytometry (C, D). Scale bars: mean ± SEM.

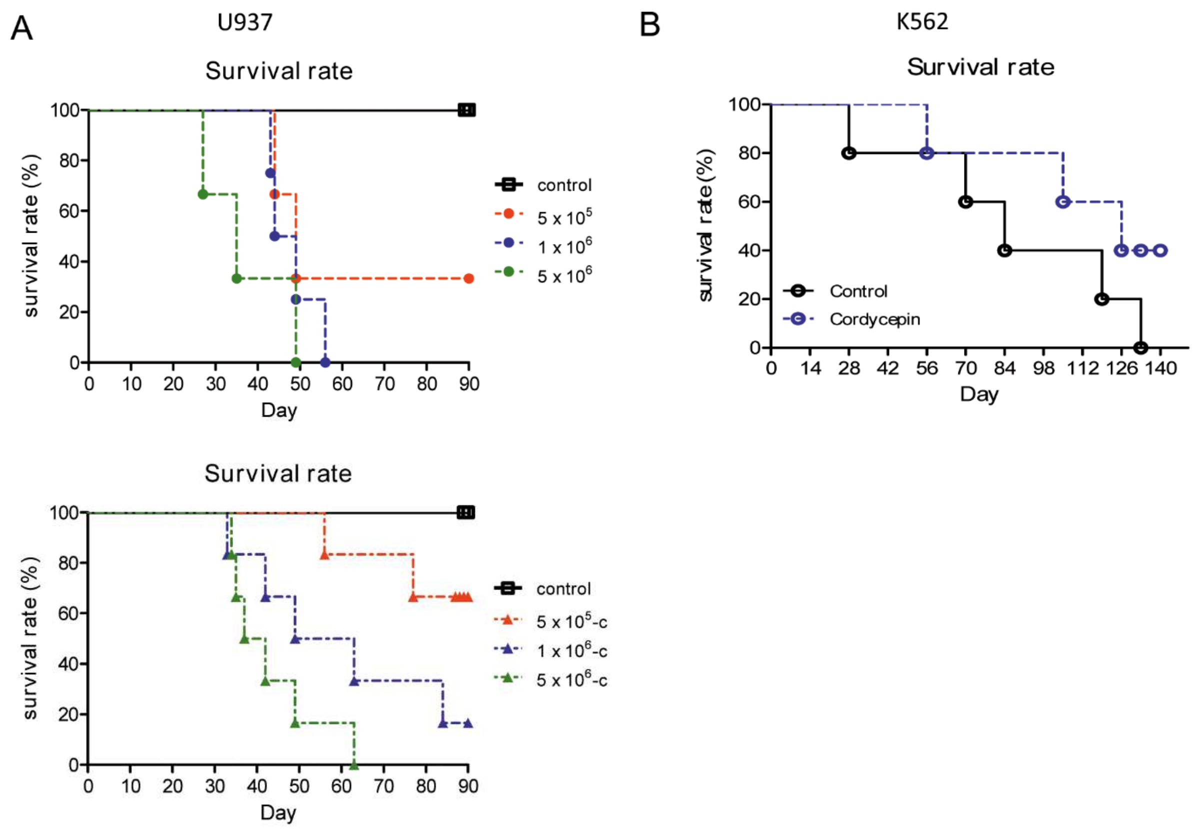

Supplementary Figure 4. Cordycepin prolongs survival of U937-inoculated mice. 5x10^5-5x10^6 U937 cells and K562 cells were pretreated with 50 μM cordycepin for 24 h and consequently intravenously injected into NOD-SCID mice. Cordycepin prolongs survival of (A) U937-inoculated (N=6) and (B) K562-inoculated mice (N=5).

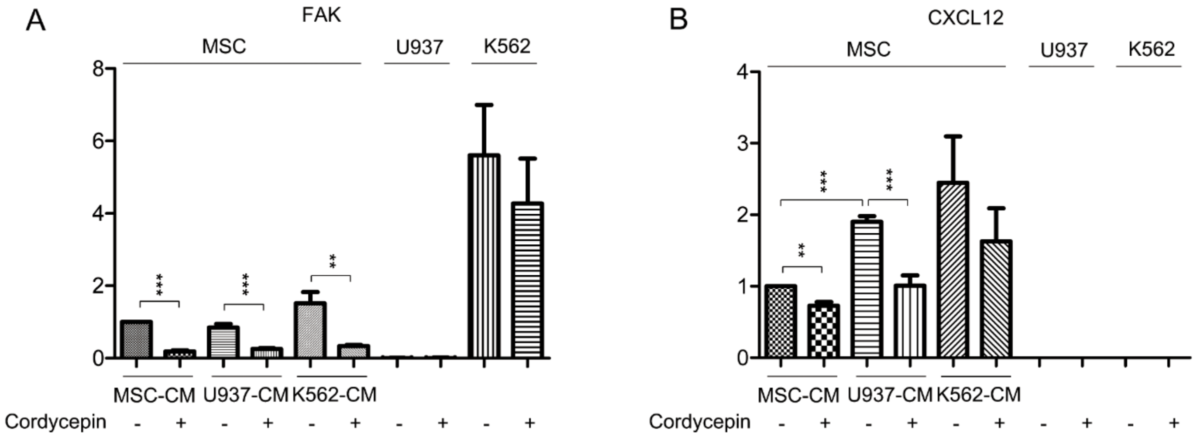

Supplementary Figure 5. Cordycepin suppresses expression of FAK and CXCL12 in leukemic CM-incubated MSCs. MSCs were incubated with MSC-CM, U937-CM and K562-CM for 48 h followed by treated with 50 μM cordycepin for additional 24 h. Expression of (**A**) FAK or (**B**) CXCL-12 in MSCs,U937 and K562 was determined by Q-PCR (N=5). Scale bars: mean ± SEM. **, *P*<0.01; ***, *P*<0.001.

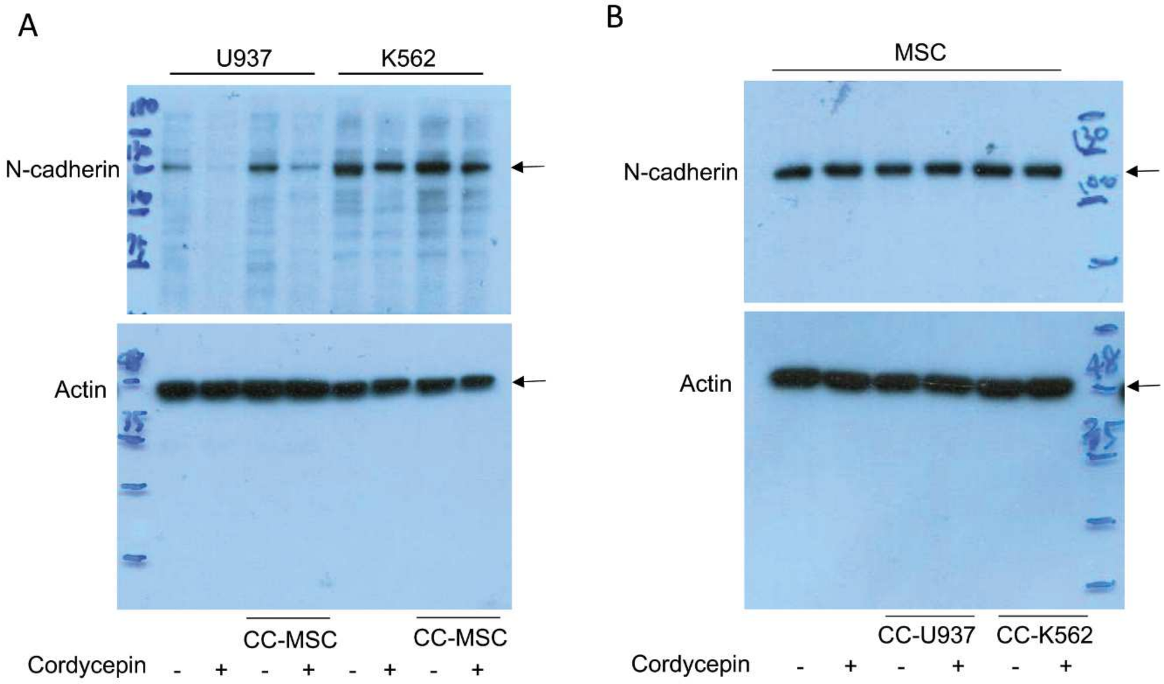

Supplementary Figure 6. The full-length blots of N-cadherin (upper panel) and Actin (lower panel) in (A) Figure 2C and (B) Figure 2D.

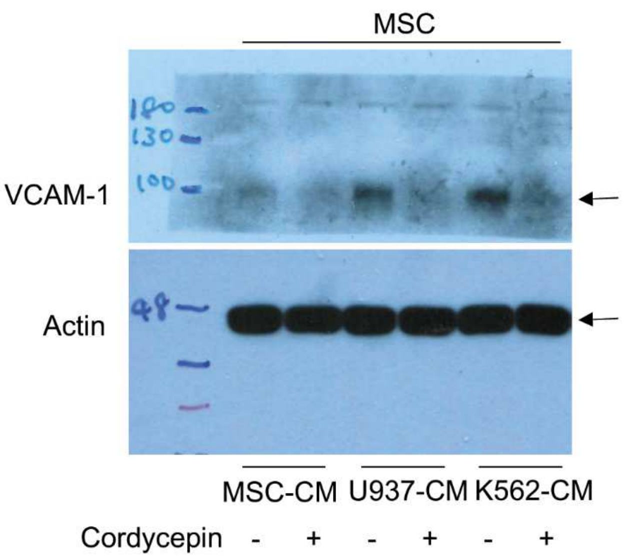

Supplementary Figure 7. The full-length blots of VCAM-1 (upper panel) and Actin (lower panel) in Figure 3B.

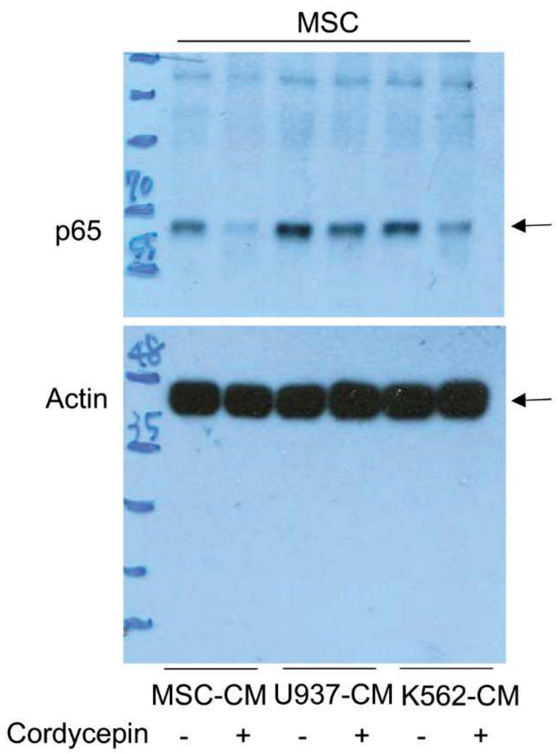

Supplementary Figure 8. The full-length blots of p65 (upper panel) and Actin (lower panel) in Figure 6B.
